# Silver Nanoparticles and Simvastatin-Loaded PLGA-Coated Hydroxyapatite/Calcium Carbonate Scaffolds

**DOI:** 10.3390/nano14201637

**Published:** 2024-10-12

**Authors:** Morena Nocchetti, Chiara Piccotti, Michela Piccinini, Silvia Caponi, Maurizio Mattarelli, Donatella Pietrella, Alessandro Di Michele, Valeria Ambrogi

**Affiliations:** 1 Dipartimento di Scienze Farmaceutiche, Università di Perugia, Via del Liceo, 1, 06123 Perugia, Italy; chiara.piccotti@yahoo.it (C.P.); michela.piccinini@studenti.unipg.it (M.P.); 2 Istituto Officina dei Materiali, National Research Council (IOM-CNR), Unit of Perugia, c/o Department of Physics and Geology, University of Perugia, Via A. Pascoli, 06123 Perugia, Italy; silvia.caponi@cnr.it; 3 Dipartimento di Fisica e Geologia, Università di Perugia, Via A. Pascoli, 06123 Perugia, Italy; maurizio.mattarelli@unipg.it (M.M.); alessandro.dimichele@unipg.it (A.D.M.); 4 Dipartimento di Medicina, Università di Perugia, Piazzale Gambuli, 1, 06132 Perugia, Italy; donatella.pietrella@unipg.it

**Keywords:** hydroxyapatite, scaffolds, simvastatin, silver nanoparticles, antimicrobial, orthopedic implants

## Abstract

The need to develop synthetic bone substitutes with structures, properties, and functions similar to bone and capable of preventing microbial infections is still an ongoing challenge. This research is focused on the preparation and characterization of three-dimensional porous scaffolds based on hydroxyapatite (HA)-functionalized calcium carbonate loaded with silver nanoparticles and simvastatin (SIMV). The scaffolds were prepared using the foam replica method, with a polyurethane (PU) sponge as a template, followed by successive polymer removal and sintering. The scaffolds were then coated with poly(lactic-co-glycolic) acid (PLGA) to improve mechanical properties and structural integrity, and loaded with silver nanoparticles and SIMV. The scaffolds were characterized by X-ray powder diffraction (XRD), field emission scanning electron microscopy (FE-SEM), ATR FT-IR, and silver and SIMV loading. Moreover, the samples were analyzed by Brillouin and Raman microscopy. Finally, in vitro bioactivity, SIMV and silver release, and antimicrobial activity against *Staphylococcus aureus* and *Staphylococcus epidermidis* were evaluated. From the Brillouin spectra, samples showed characteristics analogous to those of bone tissue. They exhibited new hydroxyapatite growth, as evidenced by SEM, and good antimicrobial activity against the tested bacteria. In conclusion, the obtained results demonstrate the potential of the scaffolds for application in bone repair.

## 1. Introduction

Bone trauma and disease, which cause severe pain and disability for many people around the world and have a high economic impact, are major clinical challenges that often require the surgical insertion of orthopedic implants [1]. These are medical devices designed to support damaged bone, replace a missing joint or bone, and augment bone tissue. Conventional methods for repairing fractures and other bone defects include autografts, allografts, xenografts, and metal implants. However, these replacement materials are far from ideal, as each of them has its own specific problems and limitations [2]. This situation stimulates extensive development of biomaterials, involving different research fields from materials science to medicine. 

Bioceramics are competitive choices for bone graft substitutes in clinical bone reconstruction due to their excellent biocompatibility and osteoconductivity/osteoinductivity. They support osteoblast adhesion, growth, and differentiation, resulting in enhanced bone formation. Selected ceramics, such as HA, are classified as “bioactive” [3]. In particular, much attention has been paid to HA, which has been considered an ideal biomaterial for bone repair for decades due to its compositional and crystallographic similarity to bioapatites, but unfortunately hinders the growth of new bone tissue because of its very poor biodegradability [4]. To overcome this problem, hybrids of hydroxyapatite and calcium carbonate/calcium oxide, which are more easily degraded, have been proposed [5,6,7]. The presence of calcium carbonate/calcium oxide improves hydroxyapatite biodegradability and facilitates the precipitation of calcium and phosphate ions. Porous forms of HA are currently generating much interest as potential bone replacement materials because porosity and pore interconnection facilitate the adhesion, proliferation, and maturation of osteoprogenitor cells, as well as adequate vascularization [8].

To improve HA performance, the combination of HA/polymer composites has been proposed. These composites offer an exceptional approach to combine the advantages of bioactive ceramics and biodegradable polymers, optimizing the physical, mechanical, and biological properties of bone implants [9]. The most frequently used synthetic polymers are biodegradable aliphatic polyesters, such as poly(glycolic acid), poly(D-lactic acid), and their copolymers PLGA [10,11]. In particular, the coating of ceramics with biocompatible and biodegradable polymers represents a promising approach that can lead to effective reinforcement while conserving the high porosity and interconnectivity of the materials, thereby encouraging the proliferation of osteoprogenitor cells. The polymer not only serves as a surface coating but also infiltrates the pores of the ceramic through processes such as coating in vacuum or centrifugation. This decreases the presence of open micropores and surface defects, reinforcing the structure and reducing brittleness by lowering the chances of crack propagation under load [12]. Therefore, bioactive and bioresorbable composites designed using polymers and HA represent an important class of biomaterials that combines the advantageous properties of their organic and inorganic components. They represent great candidates for orthopedics and bone tissue engineering owing to their numerous beneficial properties, such as tailorable degradation, biocompatibility, non-toxicity, and bioresorbability. Moreover, the polymer not only serves as a surface coating but can act as a carrier to release active agents locally and in a prolonged manner. These active agents contribute to the success of the scaffold, including antimicrobials that prevent implant-associated infections [13], or bioactive molecules able to promote new bone formation. 

The aim of the present study was to obtain scaffolds based on HA and CaCO_3_/CaO. Thus, PLGA-coated three-dimensional porous scaffolds were prepared using the replica technique, starting from hydroxyapatite-functionalized calcium carbonate (OMP). OMP is an inorganic material composed of 53% *wt*/*wt* CaCO_3_ and 47% *wt*/*wt* HA [14,15], as reported in its technical data sheet. To the best of our knowledge, only a few papers propose it as a bone filler [16,17]. The properties of these scaffolds were compared to those obtained starting from a physical mixture of HA and CaCO_3_. To confer antimicrobial activity, scaffolds were loaded with silver, whose antimicrobial properties are largely known [18,19]. Regarding osteogenic activities, SIMV, which has recently been extensively studied as an osteogenic agent, was added to the prepared scaffolds [20,21,22,23]. They were characterized by XRD, FE-SEM, and ATR FT-IR; the mechanical properties at the microscale were also evaluated using Brillouin microscopy. Finally, in vitro bioactivity, SIMV and silver release, and antimicrobial activity were evaluated.

## 2. Materials and Methods

### 2.1. Materials

Hydroxyapatite-functionalized calcium carbonate (Omyapharm^®^ 500—OG, therein named OMP) was kindly provided by Omya Italia (Milano, Italy). SIMV was purchased by Acofarma (Madrid, Spain). Hydroxyapatite, AgNO_3_, K_2_HPO_4_, NaHCO_3_, CaCO_3_, polyvinyl alcohol (PVA), and ethyl acetate were purchased from Sigma Aldrich Chemical (Milan, Italy). CaCl_2_, NaCl, KCl, MgCl_2_, Na_2_SO_4_, and tris(hydroxymethyl)aminomethane (TRIS) were purchased from Carlo Erba (Milan, Italy). Poly(lactic-coglycolic acid) (85:15, lactic acid: glycolic acid, average MW = 150,000 g/mol, hereafter, PLGA) was obtained from PolySciTech (West Lafayette, IN, USA). Polyurethane (PU) was purchased from LIGAMED Medical Produkta GmbH (Tirol, Austria). Deionized water was obtained through the reverse osmosis process using a Milli-Q system (Millipore, Rome, Italy). Other reagents and solvents were of reagent grade and were used without further purification.

### 2.2. Preparation of Scaffold Based on OMP

The OMP scaffold was prepared using the replica technique, as described by Chen et al. [24], and by using PU foams as sacrificial templates. In detail, a 2% *wt*/*wt* PVA aqueous solution was prepared by dissolving 1 g of PVA in 49 g of deionized water at 80 °C in a water bath. The slurry was prepared by adding 600 μL of PVA solution to 250 mg of OMP in a mortar. The PU foam template, cut into a cylindrical shape (diameter: 13.5 mm, height: 10 mm), was soaked in the slurry until it was fully saturated with the slurry (250 mg OMP/21 mg PU). The sample was dried in an oven at 60 °C for 12 h. After drying, the sample was sintered using the following heat temperature program: 1300 °C for 1 h, with a heating rate of 5 °C/min and a cooling rate of 10 °C/min, adapted from the literature [25,26]. The recovered scaffold, labeled as OMPs, was 6 mm in diameter and 5 mm in height, with a weight of about 180 mg. The scaffold was stored over P_2_O_5_ in a desiccator. 

As a comparison, a scaffold based on a physical mixture of HA and calcium carbonate (PM) equal to OMP in weight percentage (CaCO_3_ 53% *wt*/*wt*; HA 47% *wt*/*wt*) was prepared as described above. The sample was labeled as PMs.

### 2.3. Preparation of Ag@OMPs

The OMPs was soaked in a 5.6 × 10^−2^ M AgNO_3_ ethanol solution (mg OMPs/mL AgNO_3_ = 180/2) at room temperature for 24 h. In order to promote the diffusion of the solution into the scaffold pores, a vacuum was applied using a desiccator and maintained until all air escaped from the pores (about three minutes). All the steps were performed in the dark. The scaffold, labeled as Ag@OMPs, was recovered from the solution, washed with deionized water, and dried at 25 °C. 

### 2.4. Coating of OMPs, PMs, Ag@OMPs with PLGA

The OMPs and PMs scaffolds were dipped in the PLGA ethyl acetate solution (100 mg PLGA/1 mL ethyl acetate) under vacuum for three minutes to eliminate the air from the pores and to favor the diffusion of the polymer. Samples were obtained by repeating the procedure up to four times, and labeled as iPLGA/OMPs, where “i” indicates the number of dips and ranges from 1 to 4. The scaffolds were left to dry at 25 °C. The weight of PLGA loaded onto each scaffold, determined from the weight increase of the scaffold, is reported in Table 1. 

The Ag@OMPs was coated with PLGA by performing one immersion in PLGA ethyl acetate solution, as described above. The PLGA and Ag loading is reported in Table 1.

### 2.5. Coating of OMPs and Ag@OMPs with PLGA and SIMV 

The OMPs and Ag@OMPs were dipped once in PLGA and SIMV ethyl acetate solution (50 mg PLGA and 50 mg of SIMV in 1 mL ethyl acetate) under vacuum for three minutes. The samples (hereafter PLGA/SIMV/OMPs and PLGA/SIMV/Ag@OMPs) were left to dry at 25 °C. The weight percentage of loaded PLGA, silver, and SIMV is reported in Table 1. 

### 2.6. Bioactivity Tests in Simulated Body Fluid 

The bioactivity test on OMPs and PLGA/SIMV/Ag@OMPs was performed in Kokubo’s corrected simulated body fluid (c-SBF) [27]. The scaffolds were soaked in a volume of c-SBF such that the ratio mg of scaffold/mL c-SBF was 12, at 37 °C for 100 days. The growth of HA was assessed after 10 and 100 days after recovering the scaffolds from the solution and drying them at 25 °C.

### 2.7. In Vitro Silver and SIMV Release from PLGA/SIMV/Ag@OMPs

The PLGA/SIMV/Ag@OMPs scaffold was immersed in 100 mL of deionized water at 37 °C under mild stirring (30 rpm). At regular time intervals, 4 mL of fluid were collected and immediately replaced by the same volume of the fluid equilibrated at 37 °C ± 0.5 °C. The SIMV content was determined in 1 mL aliquots using UV-Vis spectroscopy, after proper dilution when necessary. The silver content in 1 mL aliquots was determined using ICP, after the addition of 1 mL concentrated HNO_3_ and dilution to 5 mL with deionized water. The experiment was performed in triplicate and the data were reported as an average of three measurements ± standard deviation (SD).

### 2.8. Microbial Strains

The microbial strains used in this study were Gram-positive bacteria *Staphylococcus aureus* (ATCC 29213) and *Staphylococcus epidermidis* (ATCC 12228) maintained in Mueller–Hinton agar (MHA). The day before the test, one colony was inoculated in Mueller–Hinton broth (MHB) and incubated for 24 h at 37 °C. Microbial cells were harvested by centrifugation, washed, counted by spectrophotometric analysis, and suspended in phosphate buffer solution (PBS) at a concentration of 10^8^ CFU/mL.

The scaffolds investigated for the antimicrobial were: 1PLGA/OMPs, PLGA/Ag@OMPs, PLGA/SIMV/OMPs, and PLGA/SIMV/Ag@OMPs. For these tests, cylindrical scaffolds with dimensions of diameter × height = 2 × 2 mm and weighing approximately 12 mg were prepared using the same procedure described previously.

### 2.9. Antimicrobial Susceptibility Testing 

In vitro antimicrobial susceptibility of different scaffolds was determined by the Kirby–Bauer disk diffusion method on Mueller–Hinton agar, according to the Clinical and Laboratory Standards Institute (CLSI) guidelines. Agar plates were inoculated with the microbial strains; cylindrical scaffolds were incorporated in the agar and wet with 10 μL of c-SBF. Erythromycin paper disk was used as a positive control, while 1PLGA/OMP was used as a negative control. The plates were incubated at 37 °C under aerobic conditions. The antimicrobial activity was assessed after 24 h by measuring zones of inhibition across the center of the scaffold or control disk. The diameter of the inhibition zone was measured, including the diameter of the disk, and expressed in millimeters.

### 2.10. Characterization

XRD patterns were collected using Cu-Kα radiation on a Bruker D8 Advance diffractometer (Bruker AXS GmbH, Karlsruhe, Germany), equipped with a Lynxeye XE-T detector. The long fine focus (LFF) tube was operated at 40 kV and 40 mA. To minimize preferential orientations of the microcrystals, the samples were carefully side-loaded onto a zero-background sample holder. The phase identification was performed using the Bruker DIFFRAC.EVA V5 software, equipped with the COD database. 

The Ag content in the composite was determined using a Varian Liberty inductively coupled plasma-optical emission spectrometer (ICP-OES) with axial injection. A portion of the scaffold was milled in a mortar; then, 10 mg of the obtained powder were treated with 1 mL of concentrated ammonia for 24 h, then with 1 mL of concentrated HNO_3_, and finally diluted with water. 

The SIMV content in the scaffold was determined using UV-vis spectrophotometry. A portion of the scaffold was milled in a mortar; then, 10 mg of the obtained powder were treated with 1 mL of CH_2_Cl_2_ and then properly diluted with ethanol. The amount of SIMV was determined at λ_max_ 239 nm. 

Field Emission Scanning Electron Microscopy (FE-SEM) photographs were collected using a LEO 1525 ZEISS instrument (Oberkochen, Germany) equipped with an Inlens detector and AsB (Angle selective Backscattered) detector. The samples were immobilized on a stub and coated with chromium. Elemental composition and chemical mapping were determined using a Bruker Quantax EDX (Milano, Italy).

ATR FT-IR spectra were recorded in transmittance, using a Spectrophotometer Shimadz QATR-S IR Spirit A552 (Kyoto, Japan). The wavenumber range was from 4000 to 400 cm^−1^ and the resolution was 4 cm^−1^. 

The macroscopic appearance of scaffolds after sintering and PLGA treatment was observed using a Nikon ECLIPSE 80i microscope (Tokyo, Japan) equipped with an E-Plan 4 × 0.10 ocular.

A detailed description of the custom-made setup used to perform the Brillouin and Raman micro-spectroscopy measurements is provided in the references [28,29]. 

In brief, the 532-nm single-mode solid-state laser light is focused by a microscope objective—Mitutoyo M-Plan Apo 20× (Kawasaki, Japan) with a long working distance of 20 mm and numerical aperture of 0.42. The achieved lateral optical resolution on the samples is about 2 μm. To avoid any photodamage, the laser intensity was reduced to less than 30 mW. The low-frequency part of the backscattered light is analyzed by a Raman spectrometer (monochromator Horiba iHR320 Triax, Kyoto, Japan), while the high-frequency part is analyzed by a Brillouin interferometer (tandem Fabry-Perot TFP-2 HC by JRS Scientific Instruments, Zwillikon, Switzerland). The TFP-2 HC is characterized by a high contrast of >150 dB, high luminosity of ~0.2, and a spectral resolution of 0.1 GHz, with a mirror spacing of 15 mm or more. The setup allows the correlative characterization of the mechanical and chemical properties of the materials in a non-contact and non-destructive way [30].

Brillouin spectra were obtained following a recently developed measurement and analysis procedure optimized for opaque materials [31]. The high turbidity of the investigated samples is the cause of multiple scattering, which largely affects the Brillouin spectral shape. To obtain a reliable micromechanical characterization, the data had to be appropriately corrected. 

**Figure 1:**
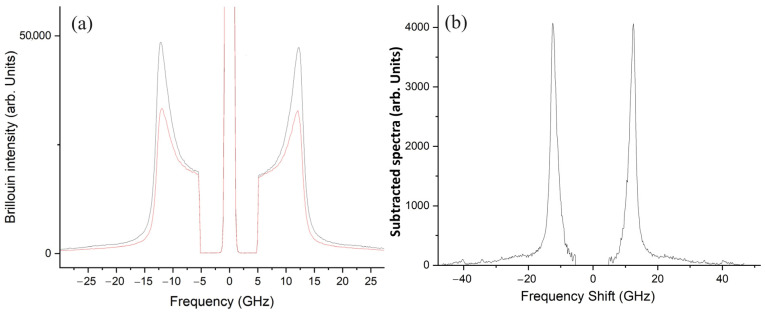
Spectra of PLGA/MPs, acquired with the same polarization as the incident laser beam: *I*_∥_ (black line) and the other one with the orthogonal polarization, *I*_⊥_ (red line), (**a**); subtracted spectrum of PLGA/MPs, (**b**).

For each material, the spectra were acquired by selecting the polarization of the scattered light, i.e., collecting light scattered with the same polarization of the incident laser beam, *I*_∥_, and the light with orthogonal polarization, *I*_⊥_. When transparent materials are investigated in a back-scattering configuration, the *I*_⊥_ is expected to be equal to zero. The multiple scattering process breaks this role, and the presence of non-negligible *I*_⊥_ confirms its important contribution to the spectra of the investigated materials. As recently demonstrated, subtracting *I*_⊥_ from *I*_∥_ once normalized in the low-frequency region, corrects the measurements, leading to the correct determination of the Brillouin frequency shift. As an example, Figure 1a reports the *I*_∥_ and *I*_⊥_ spectra, normalized on the low-frequency region, acquired for PLGA/MPs, along with the corresponding subtracted spectrum shown in Figure 1b.

## 3. Results and Discussion

### 3.1. Synthesis and Characterization of OMPs 

**Figure 2:**
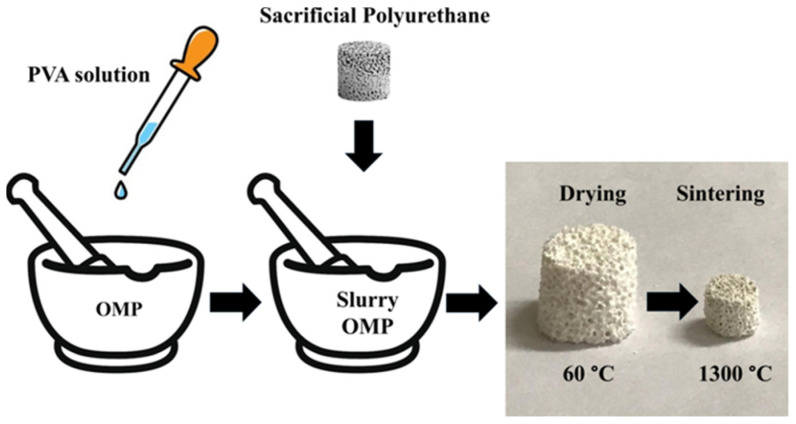
Schematic representation of the synthetic procedure to prepare the scaffold using the foam replica method.

The scaffolds were prepared following the foam replica method, as illustrated in Figure 2. The obtained scaffold (OMPs) showed size contraction while maintaining the porous structure of the plain PU. The phases present after sintering were identified by XRD.

**Figure 3:**
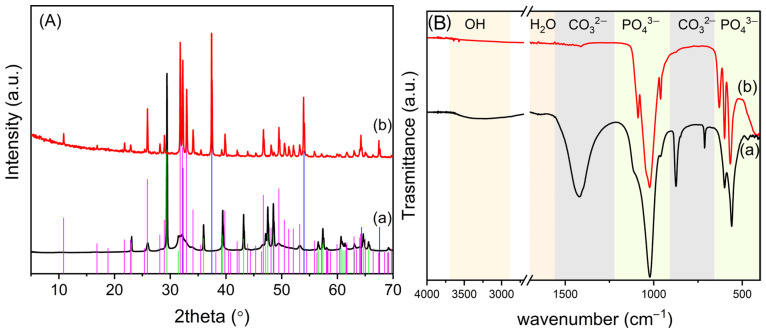
(**A**) XRD of OMP (**a**) and OMPs (**b**). In the spectra, the reflections of the following are indicated: HA (COD number: 9011097), pink line; CaCO_3_ (COD number: 9015390), green line; CaO (COD number: 1011095), blue line. (**B**) ATR FT-IR of OMP (**a**) and OMPs (**b**).

In Figure 3A, the pattern of OMPs was compared with that of untreated OMP. The XRD spectrum of OMP (Figure 3A(a)) shows the typical reflections of CaCO_3_ and HA [32]. The latter exhibits broad reflections of low intensity, suggesting poor crystallinity. After sintering (Figure 3A(b)), very sharp reflections of HA are detected, indicating its crystallization. Furthermore, the thermal decomposition of CaCO_3_ to CaO occurs.

Figure 3B compares the ATR FT-IR of the OMPs with that of the untreated material OMP; Table S1 details the wavenumbers of the adsorption bands detected in the different materials and their relative attributions. The spectrum of OMP (Figure 3B(a)) presents the typical absorption bands of HA and CaCO_3_. Specifically, the sharp bending absorption bands at 712 (ν_4_) and 875 cm^−1^ (ν_2_) and the asymmetric stretch at about 1422 cm^−1^ (ν_3_) of CO_3_^2−^ suggest the presence of the calcite phase [33]. The broad band between 2700 and 3600 cm^−1^ is ascribable to the O–H stretching, and the band at 1646 cm^−1^ to the O–H bending of adsorbed water involved in hydrogen bonds. The intense bands at 1108, 1027, and 960 cm^−1^ are due to the stretching modes of P-O, and the bands at 600 and 564 cm^−1^ are attributed to the bending modes of O-P-O [34]. The absorption bands of water and carbonate are absent after sintering (Figure 3B(b)) due to sample dehydration and decomposition of calcium carbonate to CaO, in agreement with the XRD data. Moreover, the IR spectrum of the scaffold shows bands at 3570, 3648, and 630 cm^−1^ ascribable to the stretching and bending, respectively, of the HA hydroxyl groups. In addition, the PO_4_^3−^ stretching modes in the region 900–1200 cm^−1^ appear more resolved, and the band at 1108 shifts to 1089 (ν_3a_, PO_4_^3−^), suggesting the formation of crystalline HA [35,36].

**Figure 4:**
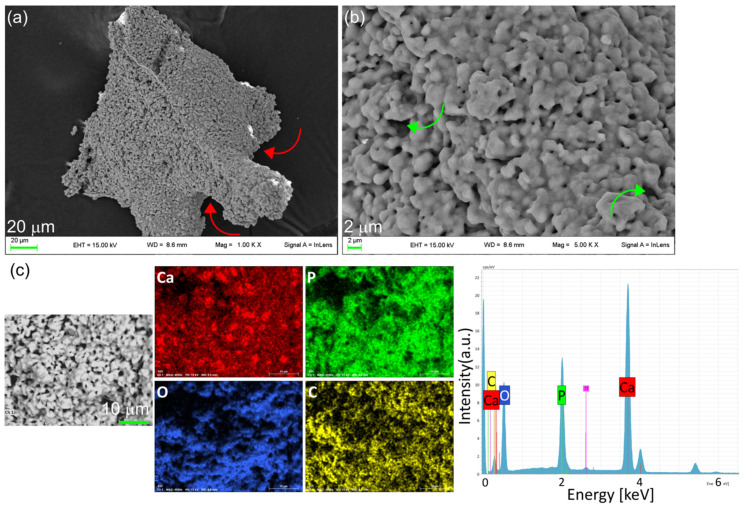
SEM images of OMPs (**a**,**b**) at different magnifications. SEM image in which porous structures are highlighted by red arrows and intrinsic interconnections are highlighted by green arrows, EDS element mappings of Ca (red), P (green), O (blue), C (yellow); and EDS spectrum of OMPs (**c**).

The morphology of the scaffold was investigated by FE-SEM, and images are reported in Figure 4a,b. The scaffold is composed of merged micrometric or submicrometric particles that form an interconnected and inherent porous structure, in addition to the macro-porosity resulting from the sacrificial template. 

The homogeneous distribution of the HA in the scaffolds is mapped out using phosphorus elemental mapping, performed by EDS (Figure 4c). EDS spectra also show the presence of a small amount of carbon, likely due to the carbonation of superficial CaO from the exposure of the scaffold to air after sintering. The homogeneous distribution of carbon is an indication of good dispersion of CaO/CaCO_3_ in the scaffold.

Scaffolds based on PM were obtained, with Figure S1 comparing the XRD and ATR FT-IR of PM with the corresponding PMs scaffold. The XRD pattern of PM shows typical HA broad reflections of low intensity (Figure S1A(a)), suggesting poor crystallinity. After sintering (Figure S1A(b)), very sharp reflections of HA and CaO were detected. The ATR FT-IR of the PM (Figure S1B(a)) shows the typical absorption bands of CaCO_3_ and HA (Table S1); after thermal treatment, the adsorption bands of PO_4_^3−^ (900–1200 cm^−1^ and 400–700 cm^−1^ region) are more resolved due to HA crystallization, as observed for OMPs. Conversely to OMPs, in PMs, residual carbonate anions remain in the scaffold, attested by the presence of the asymmetric stretch at 1410 cm^−1^ (ν_3_) of CO_3_^2−^ and its bending absorption bands at 712 (ν_4_) and 875 cm^−1^ (ν_2_).

An important property required of a biomaterial is the ability to induce the formation of bone-like apatite on its surface when bone-bonding ability is required. The in vitro bone bioactivity of both OMP and OMPs was investigated in order to study their role in the formation of new bone-like apatite from c-SBF [27]. The samples recovered from the c-SBF solution after 10 and 100 days were characterized by XRD (Figure S2) and SEM (Figure S3). After 100 days of treatment in c-SBF (Figure S2b), the typical reflections of HA are unchanged, while reflections of CaO disappear. It is very likely CaO in aqueous solution converts to Ca(OH)_2_, which then dissolves in the solution. 

Figure S3 shows SEM images of the recovered OMPs, where the formation of crystals, ascribable to new HA, is already evident on the scaffold surface after just 10 days of soaking in c-SBF. The formation of Ca(OH)_2_ in the scaffolds increases both the concentration of calcium and the pH of the medium, which rises to 10–11, despite the presence of a buffer. Such a high pH value, along with the increased concentration of calcium, promotes the precipitation of HA on the scaffold surface.

### 3.2. Functionalization of OMPs with Silver Nanoparticles

**Figure 5:**
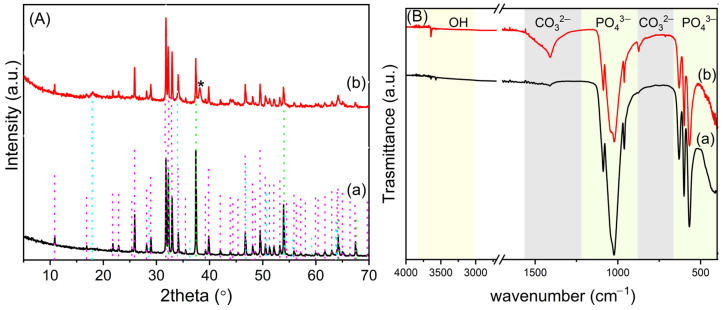
(**A**) XRD of OMPs (**a**) and Ag@OMPs (**b**). In the spectra, reflections of the following are indicated: HA (COD number: 9011097), pink line; Ca(OH)_2_ (COD number: 1008781), cyan line; CaO (COD number: 1011095), green line; Ag (COD number: 9008459) *. (**B**) ATR FT-IR of OMPs (**a**) and Ag@OMPs (**b**).

In order to confer antimicrobial activity to the scaffold, OMPs was loaded with silver by soaking in a 5.6 × 10^−2^ M AgNO_3_ ethanolic solution (Ag@OMPs). The nature of silver immobilized on the scaffold was investigated using XRD (Figure 5). The pattern of Ag@OMPs (Figure 5A(b)) was compared with that of OMPs. The XRD pattern of Ag@OMPs shows typical reflections of HA, which remain unchanged in comparison with OMPs, along with a new reflection ascribable to the (111) planes of the cubic phase of metallic Ag. The CaO phase exhibits reflections of lower intensity compared to OMPs, owing to the water present in ethanol that partially converts CaO into Ca(OH)_2_. The formation of silver nanoparticles can be due to the reduction of silver ions to metallic silver by ethanol, a reduction that is favored in alkaline mediums [37]. The silver content was 4.1% *wt*/*wt*. Ag@OMPs was characterized using ATR FT-IR (Figure 5B) in order to study the possible changes in OMPs upon AgNPs immobilization. The Ag@OMPs spectrum is similar to that of OMPs, except for the appearance of the absorption bands of carbonate. The presence of carbonate can be attributed to partial carbonation resulting from the acid–base reaction between Ca(OH)_2_ and atmospheric CO_2_. 

**Figure 6:**
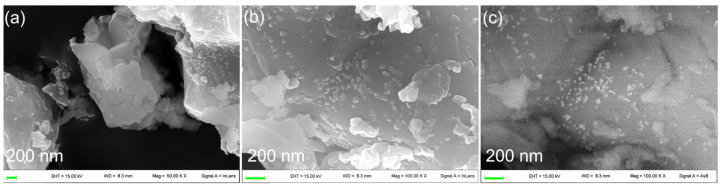
SEM images of Ag@OMPs (**a**–**c**) at different magnitudes and collected with secondary electron (**a**,**b**) and back-scattered (**c**) detector.

The morphology and the dimensions of metallic silver in Ag@OMPs were investigated using SEM (Figure 6). The images display a uniform distribution of 10–20 nm nanoparticles ascribable to silver, as they appear as bright particles when the back-scattered detector is used.

### 3.3. Fabrication of PLGA/OMPs and PLGA/SIMV/Ag@OMPs Composites 

**Figure 7:**
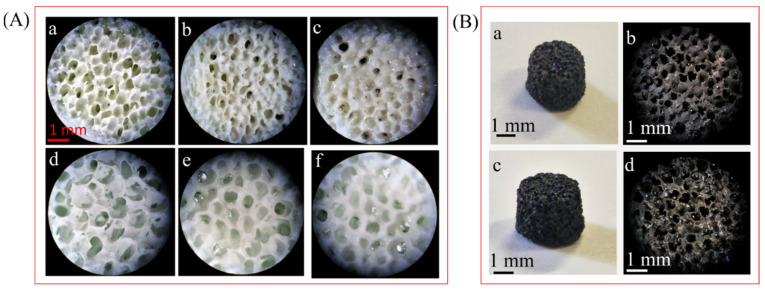
(**A**) Optical microscope images of OMPs (**a**) and PMs (**d**) after dipping in PLGA solution once (**b**,**e**) and twice (**c**,**f**). (**B**) Digital and optical microscope images of Ag@OMPs (**a**,**b**) and PLGA/SIMV/Ag@OMPs (**c**,**d**).

To improve the mechanical properties of the OMPs, the scaffolds were coated with PLGA by soaking them in a polymer solution. The immersion was repeated four times, and the scaffolds obtained were analyzed using an optical microscope. Figure 7A(a–c) shows the optical microscope images of OMPs, 1PLGA/OMPs, and 2PLGA/OMPs. OMPs is characterized by pores with a diameter of 87.6 ± 29.4 μm. After one immersion in PLGA, the porous structure is maintained, while after the second immersion, the pores of 2PLGA/OMPs are mostly closed. In light of these findings, 1PLGA/OMPs was chosen for subsequent experiments. For comparison, PMs was also submitted to the PLGA coating, and its image is shown in Figure 7A(d). The picture of PMs displays a regular porous structure, with pores of an average diameter of 127.6 ± 23.5 μm. Similar to OMPs, the ideal coverage is reached after one immersion in PLGA. Nevertheless, the larger pores result in a less resistant structure compared with OMPs. The scaffolds based on PMs, despite being coated with PLGA, are very brittle and the structure collapses during handling. Thus, it was not possible to submit them to further experiments. Although OMPs and PMs have the same composition, they differ in the interactions between the two components, HA and CaCO_3_. In particular, OMPs is obtained by growing HA on CaCO_3_, forming chemical connections that increase structural integrity.

The coating of the scaffold with PLGA was applied to Ag@OMPs not only to enhance the structural integrity but also to load SIMV, a molecule able to improve bone regeneration. Figure 7B shows digital and optical microscope images of Ag@OMPs and PLGA/SIMV/Ag@OMPs. The scaffolds are dark brown colored due to the presence of silver nanoparticles, and their porous structure is retained after immersion in the PLGA/SIMV solution. The SIMV content, determined using UV-Vis spectroscopy, was 6.7% *wt*/*wt*.

**Figure 8:**
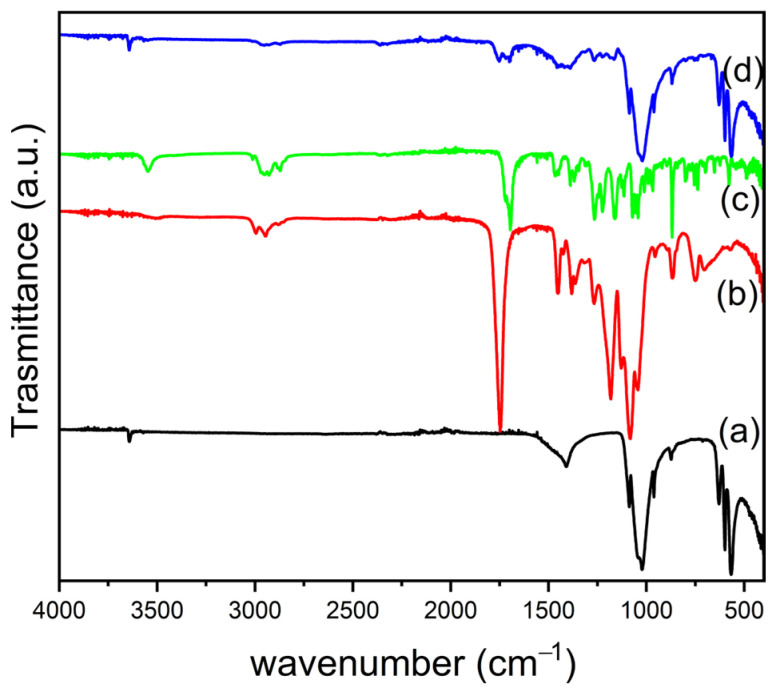
ATR FT-IR of Ag@OMPs (**a**), PLGA (**b**), SIMV (**c**), and PLGA/SIMV/Ag@OMPs (**d**).

The ATR FT-IR spectra (Figure 8) were collected for Ag@OMPs, neat PLGA, SIMV, and PLGA/SIMV/Ag@OMPs. The spectrum of the final composite is a combination of the spectra of the individual components, indicating that during the establishment of the final formulation, no component was changed or omitted. For the sake of clarity, the main bands of PLGA and SIMV are discussed. The IR spectrum of PLGA shows an intense band at 1748 cm^−1^, attributable to the stretch of carbonyl groups, as well as the stretching bands of C–H groups in the region between 2950 and 3000 cm^−1^. The bands at 1447 and 1377 cm^−1^ correspond to the bending vibrations of CH_2_ and CH(CH_3_) [38]. The IR spectrum of the neat SIMV shows the characteristic peaks at 3550 cm^−1^ (free O–H stretching vibration), 2950–3000 cm^−1^ (aliphatic C–H stretching vibrations), 1717 and 1693 cm^−1^ (stretching vibration of C=O for ester and lactone), 1268 cm^−1^ (lactone –C–O–C stretching vibration). The composites were also characterized by XRD and μ-Raman (Figure S4).

#### 3.3.1. Mechanical Properties

Brillouin microscopy measures the mechanical properties at the microscale by enabling the extraction of the local longitudinal elastic modulus of the investigated material from the frequency position of the Brillouin peak [39]. In heterogeneous samples such as bones, Brillouin spectroscopy has revealed the co-presence of at least two main spectroscopic features [40], which are assigned to the coexistence of soft and hard micro-structures that are simultaneously present in the bone composition. To ensure the correct tissue functionality, the presence of a soft part associated with the organic component of the tissue (collagen fibers, cells, and Haverzian and Volkmann’s canals) guarantees the exchange of nutrients and supports bone regeneration. This soft component is interspersed with the hard structures associated with the mineralized fibers, providing the bone tissue with rigidity and the ability to withstand stress [41].

**Figure 9:**
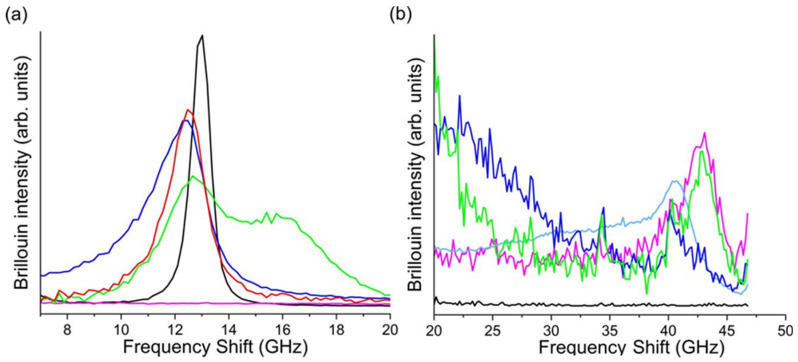
Brillouin spectra at low (**a**) and high-frequency region (**b**) of OMPs (magenta line), PLGA (black line), PLGA/OMPs (red line), PLGA/MPs (blue line), Ag@OMPs (cyan line), PLGA/SIMV/OMPs (green line).

It is worth noticing that the Brillouin spectrum of the here-developed scaffolds also presents two components, resembling the features of bone tissue. The harder part consists of the OMP structure, displaying a Brillouin peak around 43 GHz (Figure 9b). The hard component in PLGA/MPs exhibits low intensity and falls at a lower frequency, which is in agreement with the brittle behavior observed at the macroscopic scale compared with OMPs. The soft part is composed of PLGA, showing a characteristic peak around 13 GHz (Figure 9a).

In the scaffolds functionalized with silver and coated with PLGA/SIMV (Ag@OMPs, PLGA/SIMV/OMPs), the elastic characteristics in the high-frequency region, typical of bare OMPs, are maintained. In the low-frequency range, small variations can be seen for the PLGA/MPs and PLGA/OMPs. Upon adding SIMV to PLGA (PLGA/SIMV/OMPs), a new peak appears at about 16 GHz, indicating the stiffening of a portion of the polymer, likely due to SIMV/PLGA interactions.

#### 3.3.2. In Vitro Bioactivity Properties and Silver and SIMV Release of PLGA/SIMV/Ag@OMPs

**Figure 10:**
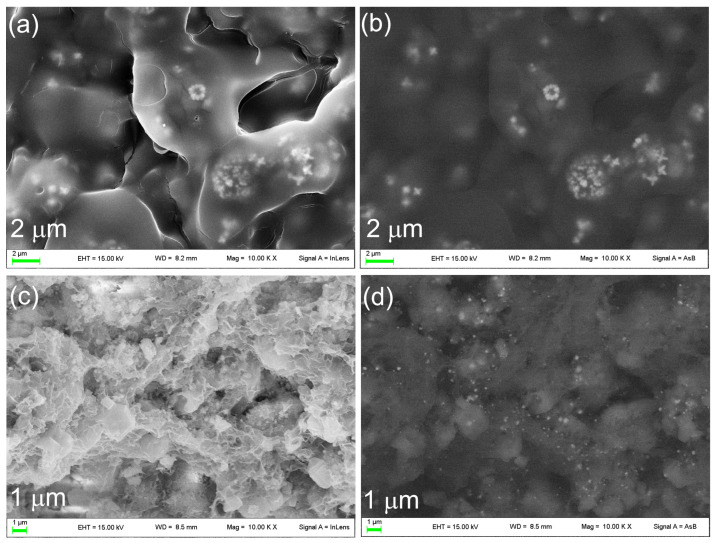
SEM images of PLGA/SIMV/Ag@OMPs before (**a**,**b**) and after 10 days in c-SBF (**c**,**d**) collected with secondary electron (**a**,**c**) and back-scattered (**b**,**d**) detector.

The bioactivity of PLGA/SIMV/Ag@OMPs was evaluated in c-SBF. The formation of new HA was investigated using SEM. Figure 10 compares the images of PLGA/SIMV/Ag@OMPs before and after soaking for 10 days in c-SBF. The PLGA/SIMV/Ag@OMPs appears covered in the polymeric film under which silver nanoparticles are detected (Figure 10a,b). After immersion in c-SBF, the polymer appears degraded and is substituted by a quite uniform layer of bone-like HA (Figure 10c,d). The presence of silver is noticeable, suggesting that even if it is released, it undergoes re-precipitation in the form of a poorly soluble salt due to the presence of the anions in c-SBF.

**Figure 11:**
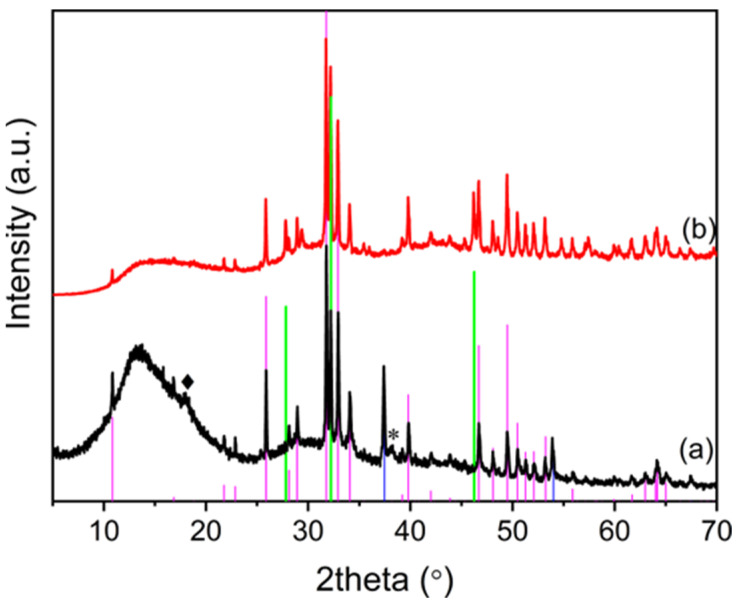
XRD of PLGA/SIMV/Ag@OMPs before (**a**) and after 30 days in c-SBF (**b**). In the spectra, the reflections of the following are indicated: HA (COD number: 9011097), pink line; CaO (COD number: 1011095), blue line; PLGA ◆; Ag *; AgCl (COD number: 9008597) green line.

The scaffold PLGA/SIMV/Ag@OMPs was characterized using XRD in order to identify the phases present after 30 days in c-SBF and to compare them with the starting PLGA/SIMV/Ag@OMPs. After the treatment in c-SBF (Figure 11b), no modifications on the typical reflections of HA were detected, while the reflections of CaO disappeared, most likely converting into new HA. Moreover, the typical reflections of AgCl indicate the re-precipitation of the released silver.

**Figure 12:**
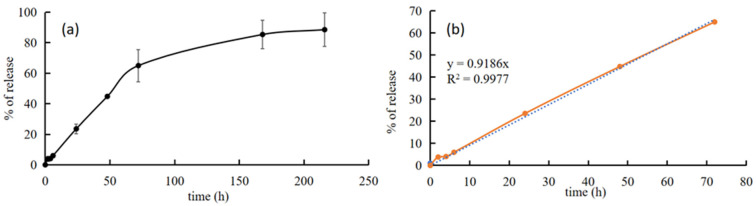
Release profile of SIMV from PLGA/SIMV/Ag@OMPs in aqueous media at 37 °C for 9 days (**a**) and kinetic release curve according to zero-order kinetics (3 days) with relative equation and correlation coefficient (**b**).

SIMV release from PLGA/SIMV/Ag@OMPs was performed in deionized water and evaluated until a plateau was reached (9 days). Results are shown in Figure 12. SIMV release was gradual and slow, and no burst effect was detected. After 3 days (72 h), the percentage of released SIMV was ca. 65%, and then the release continued slower and it was almost complete (85%) after one week (172 h).

It should be highlighted that drug release during the first 3 days (65% of SIMV release) followed zero-order kinetics (R^2^ = 0.9988). This means that this system is able to provide a constant rate of release for a predictable period of time, independent of the concentration of the drug in the system. The drug is incorporated into the PLGA and released in the surrounding environment as the polymer erodes. The degradation of PLGA is sensitive to the pH of the environment, with PLGA degrading faster in a highly alkaline buffer [42]. The pH of the release fluid was monitored during the test and was ca. 8 after 24 h, increasing successively to 11, which favored polymer erosion due to the environmental conditions. The zero-order release obtained during the first 3 days can be explained by the erosion process of the polymer coating of the scaffold and the subsequent drug release. In fact, zero-order release requires that the erosion process be confined to the surface of the solid device and that the mobility of the drug is low enough that diffusion is negligible [43]. After the third day, the drug release was slower, so the diffusion of the drug along the pores of the scaffold must also be considered.

Silver release, determined by ICP, was much low (0.51% after 24 h) and showed fluctuations. This can be explained both by the alkaline environment of the release fluid and by the exposure of the scaffold surface (and therefore by HA). The increase of the environmental pH, in which silver is not soluble [44], is due to CaO presence. The solubilisation of the PLGA coating allows HA to react with silver ions. It should be highlighted that silver release under in vivo conditions could be different because of the presence of physiological buffers.

#### 3.3.3. Antimicrobial Activity

A very large proportion of all implant-related infections are caused by staphylococci, among which *Staphylococcus aureus* and *Staphylococcus epidermidis* are components of the skin microbiota [45].

**Figure 13:**
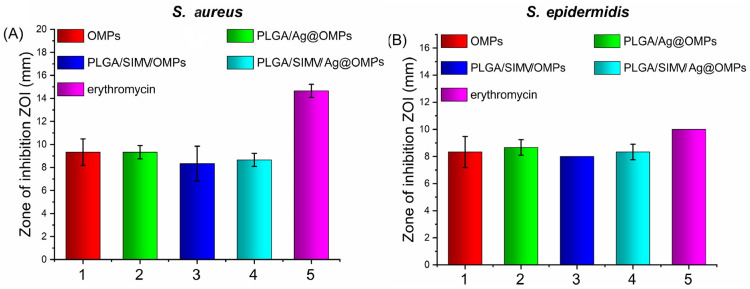
Antimicrobial activity of scaffolds towards *S. aureus* (**A**) and *S. epidermidis* (**B**). Results are reported as the mean values ± standard deviation of inhibition zone determined by the Kirby–Bauer assay of three independent experiments. The control was erythromycin.

Thus, the antimicrobial activity of all prepared scaffolds was evaluated against these bacteria strains, which typically infect implants. The antimicrobial activities of scaffolds were evaluated by the Kirby–Bauer test, and the results are reported in Figure 13. Interesting to note is that all scaffolds showed antimicrobial activity, including those free of silver. 

Silver antimicrobial activity is well documented in the literature, thus the activity of PLGA/SIMV/Ag@OMPs and PLGA/Ag@OMPs was expected. The activity of silver-free scaffolds has already been hypothesized for bioglasses. Moreover, in recent years, there has been increasing interest in the potential antibacterial properties of bioactive glass. It has been hypothesized that the antibacterial activities of bioglass are due to the increase in local pH following the exchange of sodium ions with protons in body fluids. The alkaline environment constitutes a stress for the bacteria, which respond by changing their morphology and ultrastructure, modifying the expression pattern of numerous genes and proteins [46]. When immersed in c-SBF and deionized water, the herein-described scaffolds generate an alkaline environment with pH values of ca. 8 after 24 h, increasing successively up to 11. This increase is due to CaO, which, in the presence of water, is converted to Ca(OH)_2_, resulting in a consequent increase in pH. These pH values are maintained throughout all the tests (bioactivity test and SIMV release test), despite the PLGA hydrolysis, which usually induces a pH decrease. 

## 4. Conclusions

The goal of this research was to obtain three-dimensional porous scaffolds for supporting bone regeneration and growth, preventing implant-related infections.

Scaffolds based on hydroxyapatite-functionalized calcium carbonate were successfully prepared using the foam replica method, followed by coating with the polymer PLGA. Moreover, scaffolds containing silver nanoparticles and/or SIMV were also prepared successfully. The obtained scaffolds showed macro-porosity and an interconnected inherent porous structure, with silver nanoparticles quite homogeneously dispersed on the scaffold surface. Additionally, there was an improvement in mechanical properties following the coating with PLGA, as well as good bioactive properties for both PLGA-free and coated scaffolds. The scaffolds also demonstrated good antimicrobial activity against the tested bacteria, *Staphylococcus aureus* and *Staphylococcus epidermidis*.

In conclusion, the objectives that were set have been achieved, and the scaffolds described here deserve to be evaluated for their cytotoxicity, as well as their ability to affect bone cell metabolism and induce osteogenesis. After these preliminary in vitro studies, a clinical evaluation will be planned.

## Figures and Tables

**Table 1:** Weight percentage of PLGA, silver, and SIMV in the indicated scaffolds (g/100 g of final scaffold).

Sample ^a^	PLGA (wt%)	Ag (wt%)	SIMV (wt%)
1PLGA/OMPs	9.3	-	
2PLGA/OMPs	15.0	-	
3PLGA/OMPs	18.0	-	
4PLGA/OMPs	24.1	-	
PLGA/Ag@OMPs	7.4	4.3	-
PLGA/SIMV/OMPs	12.9	-	6.4
PLGA/SIMV/Ag@OMPs	12.6	4.1	6.7

^a^ The numerical prefixes that precede the names of the scaffolds indicate the number of immersions of the scaffold in PLGA solution. Ag, SIMV, PLGA and OMPs stand for silver, simvastatin, poly(lactic-coglycolic acid), and OMP-based scaffold, respectively.

## Data Availability

Data are contained within the article and Supplementary Materials.

## Supplementary Materials

The following supporting information can be downloaded at: https://www.mdpi.com/article/10.3390/nano14201637/s1, Table S1: Wavenumber of the adsorption bands detected in the ATR FT-IR spectra of OMP, OMPs, PM, and PMs and the relative attributions. Figure S1: XRD and ATR FT-IR of PM and PMs. Figure S2: XRD of OMPs and OMPs after 100 days in c-SBF. Figure S3: SEM images of OMPs at different magnifications after 10 days in c-SBF. Figure S4: XRD of OMPs, PLGA/OMPs, and PLGA/SIMV/Ag@OMPs; μ-Raman spectra of pure OMPs, PLGA, SIMV, and of PLGA/SIMV/Ag@OMPs; structural formula of SIMV. Refs. [38,47,48,49,50,51,52,53,54,55] are cited in the Supplementary Materials.
