# Supplementary material for: Silver Nanoparticles and Simvastatin-Loaded PLGA-Coated Hydroxyapatite/Calcium Carbonate Scaffolds
Source: Nanomaterials (Basel). 2024 Oct 12;14(20):1637. doi: 10.3390/nano14201637 (PMC11510553; doi:10.3390/nano14201637)
Supplement: Supplementary file 1 [file nanomaterials-14-01637-s001.zip › nanomaterials-3227062-supplementary.pdf]

Supplemental Information

Silver Nanoparticles and Simvastatin-Loaded PLGA-Coated Hydroxyapatite/Calcium Carbonate Scaffolds

Morena Nocchetti [1,*], Chiara Piccotti [1], Michela Piccinini [1], Silvia Caponi [2], Maurizio Mattarelli [3], Donatella Pietrella [4], Alessandro Di Michele [3] and Valeria Ambrogi [1,*]

[1] Dipartimento di Scienze Farmaceutiche, Università di Perugia, Via del Liceo, 1, 06123 Perugia, Italy; chiara.piccotti@yahoo.it (C.P.); michela.piccinini@studenti.unipg.it (M.P.)

[2] Istituto Officina dei Materiali, National Research Council (IOM-CNR), Unit of Perugia, c/o Department of Physics and Geology, University of Perugia, Via A. Pascoli, 06123 Perugia, Italy; silvia.caponi@cnr.it

[3] Dipartimento di Fisica e Geologia, Università di Perugia, Via A. Pascoli, 06123 Perugia, Italy; maurizio.mattarelli@unipg.it (M.M.); alessandro.dimichele@unipg.it (A.D.M.)

[4] Dipartimento di Medicina, Università di Perugia, Piazzale Gambuli, 1, 06132 Perugia, Italy; donatella.pietrella@unipg.it

* Correspondence: morena.nocchetti@unipg.it (M.N.); valeria.ambrogi@unipg.it (V.A.)

Table S1. Wavenumber of the adsorption bands detected in the ATR FT-IR spectra of different materials (Figure 3 (B) and Figure S1 (B)) and the relative attributions.

Wavenumber (cm^{-1})	Sample	Attribution
3570/3648	OMPs/ PMs	Stretching O-H of HA
3600-2700	OMP	Stretching O-H of water
1646	OMP	Bending O-H of water
1422	OMP	Stretching asym. (ν_3) CO_3^{2-}
1410	MP; PMs	Stretching asym. (ν_3) CO_3^{2-}
1108	OMP	Stretching asym (ν_{3a}) PO_4^{3-} (P–O bond)
1089	OMPs; PM; PMs;	Stretching asym (ν_{3a}) PO_4^{3-} (P–O bond)
1040	PMs;	Stretching asym (ν_{3c}) PO_4^{3-} (P–O bond)
1027	OMP; OMPs; PMs	Stretching asym (ν_{3c}) PO_4^{3-} (P–O bond)
960	OMP; OMPs; PM; PMs;	Stretching sym (ν_1) PO_4^{3-} (P–O bond)
875	OMP; PM; PMs;	Bending (ν_2) CO_3^{2-}
712	OMP; PM; PMs	Bending (ν_4) CO_3^{2-}
630	OMPs; PMs;	Bending O-H of HA
600	OMP; OMPs; PMs;	Bending (ν_{4a}) PO_4^{3-} (O-P–O bond)
564	OMP; OMPs; PMs;	Bending (ν_{4b}) PO_4^{3-} (O-P–O bond)

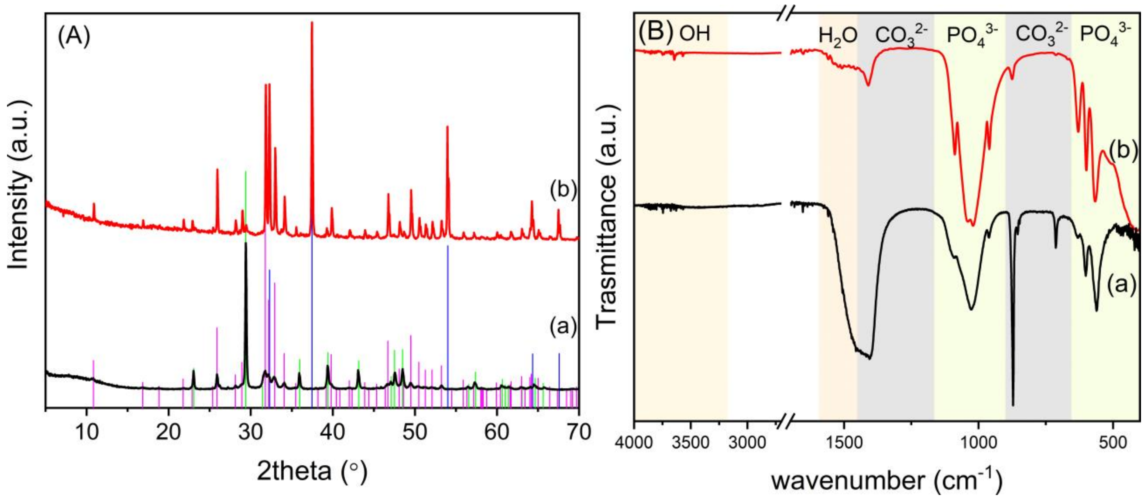

Figure S1. (A) XRD of PM (a) and PMs (b), in the spectrum are indicated the reflections of: HA (COD number: 9011097), pink line; $CaCO_3$ (COD number: 9015390), green line; CaO (COD number: 1011095), blue line. (B) ATR FT-IR of PM (a) and PMs (b).

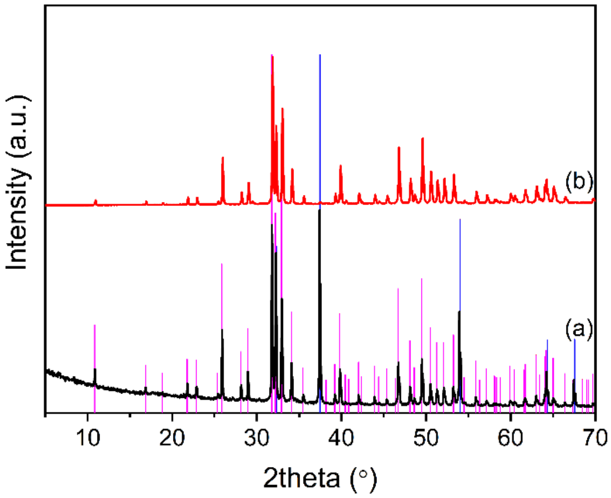

Figure S2. XRD of OMPs (a) and OMPs after 100 days in SBF (b). In the spectra are indicated the reflections of: HA (COD number: 9011097), pink line; CaO (COD number: 1011095), blue line.

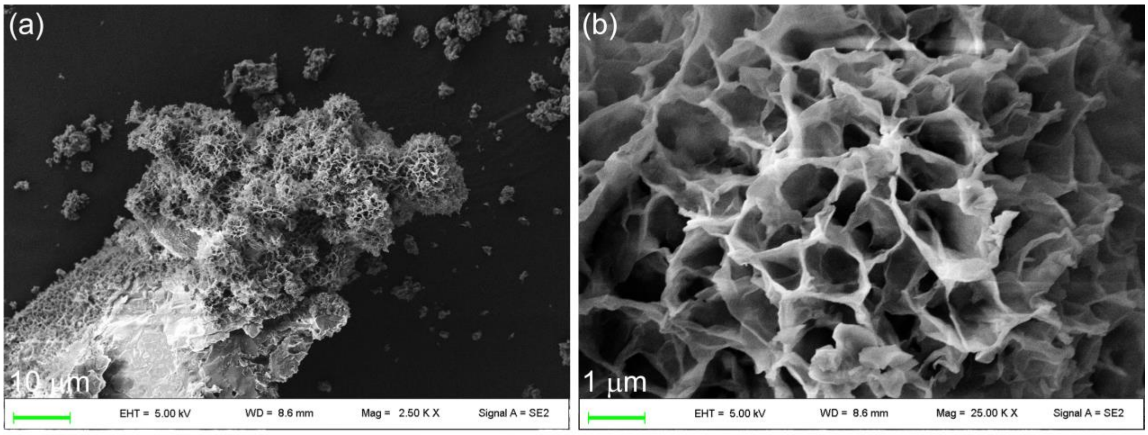

Figure S3. SEM images of OMPs (a,b) at different magnifications after 10 days in SBF.

Figure S4 shows the XRD of PLGA/SIMV/Ag@OMPs in comparison with those of OMPs and PLGA/OMPs. The pattern of PLGA/SIMV/Ag@OMPs exhibits the typical reflections of HA, the intensity of the CaO reflections was significantly reduced due to the presence of water in the solvent used to load silver (ethanol at 95%) and to solubilize PLGA (ethyl acetate). Furthermore, a reflection of the cubic phase of metallic silver and broad reflection with low intensity of PLGA [1] were detected.

The PLGA/SIMV/Ag@OMPs and the single components (OMPs, PLGA and SIMV) were also characterized by μ-Raman spectroscopy (Figure S4), a non-destructive technique, that allows to identify the chemical composition and the molecular organization in a wide range of materials [2-4]. As far the μ-Raman spectrum of pure OMPs, in the region 350-1250 cm^{-1} the strong peak at 961

cm^{-1} is due to the symmetric stretching (ν_1) of PO_4^{-3} tetrahedra. The band centered at 1044 cm^{-1} can be assigned to the asymmetric (ν_3) P-O stretching while the bands centered at 424 and 578 cm^{-1} correspond to the O-P-O bending mode (ν_2) and the rotational modes mode (ν_4) of the PO_4^{-3}, respectively [5]. The Raman shift observed for the internal modes of the PO_4^{3-}, and the frequency gap between ν_2 and ν_4 mode of 154 cm^{-1}, are typical of HA structure [6]. The bands at 3573 cm^{-1} and 3615 cm^{-1} can be assigned to the O-H stretching mode of the hydroxyl group present in the HA structure [7], and the adsorption band at 353 cm^{-1} corresponds to the translational mode of the hydroxyl group. The small shoulder at 1077 cm^{-1} is due to traces of CO_3^{2-} indicating a partial carbonation of the apatite or CaO. The spectrum of the neat PLGA displays the vibrational modes both of lactic acid and the glycolic acid segments; the band at 1463 cm^{-1} is assigned to the CH_2 stretching from glycolide segments, the bands at 2892, 2950 and 3007 cm^{-1} are due to the $-CH_2$ and $-CH_3$ stretching of glycolide and lactide segments, respectively. The vibration mode at 1770 cm^{-1} is ascribable to the stretching of C=O [8]. The 875 cm^{-1} band is assigned to the C−COO stretch vibration of lactide segments [9]. The micro-Raman spectrum of SIMV shows the stretch vibration of the C=O group of the ester in the aliphatic chain of a SIMV molecule and $-CH_2$ and $-CH_3$ stretching in the 2850-3010 cm^{-1} [10]. The spectrum of the PLGA/SIMV/Ag@OMPs shows the typical vibration modes of the single components without considerable shifts.

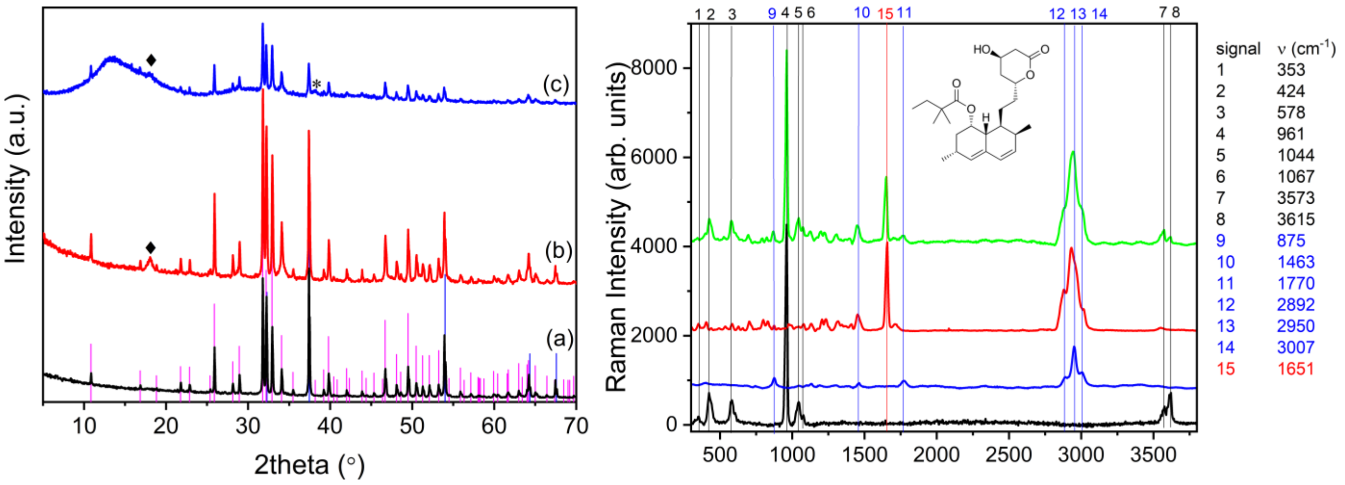

Figure S4. Left: XRD of OMPs (a), PLGA/OMPs (b) and PLGA/SIMV/Ag@OMPs (c). In the spectra are indicated the reflections of: HA (COD number: 9011097), pink line; CaO (COD number: 1011095), blue line; PLGA ♦; Ag *. Right: μ-Raman spectra of pure OMPs (black line), PLGA (blue line), SIMV (red line) and of PLGA/SIMV/Ag@OMPs (green line). The structural formula of SIMV is also reported.

References

[1] Zhang, Z.; Wang, X.; Zhu, R.; Wang, Y.; Li, B.; Ma, Y.; Yin, Y. Synthesis and characterization of serial random and block-copolymers based on lactide and glycolide. *Polym. Sci. Ser. B Chem.* **2016,** *58*, 720-729.

[2] Donnadio, A.; Bini, M.; Centracchio, C.; Mattarelli, M.; Caponi, S.; Ambrogi, V.; Pietrella, D.; Di Michele, A.; Vivani, R.; Nocchetti, M. Bioinspired reactive interfaces based on layered double hydroxides-Zn rich hydroxyapatite with antibacterial activity. *ACS Biomater. Sci. Eng* **2021**, *7*, 1361-1373.

[3] Caponi, S.; Liguori, L.; Giugliarelli, A.; Mattarelli, M.; Morresi, A.; Sassi, P.; Urbanelli, L.; Musio, C. Raman micro-spectroscopy: A powerful tool for the monitoring of dynamic supramolecular changes in living cells. *Biophys. Chem.* **2013,** *12,* 58-63.

[4] Cazzolli, G.; Caponi, S.; Defant, A.; Gambi, C.M.C.; Marchetti, S.; Mattarelli, M.; Montagna, M.; Rossi, B.; Rossi, F.; Viliani, G. Aggregation processes in micellar solutions: a Raman study. *J. Raman Spectrosc.* **2012**, *43*, 1877-1883.

[5] Koutsopoulos, S. Synthesis and characterization of hydroxyapatite crystals: A review study on the analytical methods. *J. Biomed. Mater. Res.* **2002**, *62*, 600-612.

[6] Cusco, R.; Guitian, F.; de Aza, S.; Artus, L. Differentiation between Hydroxyapatite and β-Tricalcium Phosphate by Means of μ-Raman Spectroscopy. *J. Eur. Ceram. Soc.* **1998,** *18*, 1301-1305.

[7] Khan, A.F.; Awais, M.; Khan, A.S.; Tabassum, S.; Chaudhry, A.A.; Rehman, I.U. Raman Spectroscopy of Natural Bone and Synthetic Apatites. *Appl. Spectrosc. Rev.* **2013**, *48*, 329-355.

[8] Biswal, A.K.; Hariprasad, P.; Saha, S. Efficient and prolonged antibacterial activity from porous PLGA microparticles and their application in food preservation. *Mater. Sci. Eng.* **2020,** *C 108*, 110496.

[9] van Apeldoorn, A.A.; Aksenov, Y.; Stigter, M.; Hofland, I.; de Bruijn, J.D.; Koerten, H.K.; Otto, C.; Greve, J.; van Blitterswijk, C.A. Parallel high-resolution confocal Raman SEM analysis of inorganic and organic bone matrix constituents. *J. R. Soc. Interface* **2015,** *2,* 39-45.

[10] Heilmann, M.T.; Simoes, R.G.; Bernardes, C.E.; Ramisch, Y.; Bienert, R.; Röllig, M.; Emmerling, F.; Minas da Piedade, M.E. Real-Time In situ XRD Study of Simvastatin Crystallization in Levitated Droplets. *Cryst. Growth Des.* **2021**, *21*, 4665-4673.
